# An Assessment of the Stability of the Canine Oral Microbiota After Probiotic Administration in Healthy Dogs Over Time

**DOI:** 10.3389/fvets.2020.00616

**Published:** 2020-09-11

**Authors:** Sara E. Bell, Andrea K. Nash, Brian M. Zanghi, Cynthia M. Otto, Erin B. Perry

**Affiliations:** ^1^Department of Pathobiology, University of Pennsylvania School of Veterinary Medicine, Philadelphia, PA, United States; ^2^Penn Vet Working Dog Center, University of Pennsylvania School of Veterinary Medicine, Philadelphia, PA, United States; ^3^Nestle Purina Research, St. Louis, MO, United States; ^4^Department of Clinical Sciences and Advanced Medicine, University of Pennsylvania School of Veterinary Medicine, Philadelphia, PA, United States; ^5^Department of Animal Science, Food and Nutrition, College of Agricultural Science, Southern Illinois University, Carbonale, IL, United States

**Keywords:** oral microbiota, canine, probiotic, stability, diet

## Abstract

The administration of an oral probiotic has been demonstrated to impact oral microbial diversity in humans but has not been examined in canines. The objective of this study was to test the hypothesis that oral probiotic administration would impact the oral microbiota of canines compared to control. Working canines in training (*n* = 13) were assigned to Test or Control groups and acclimated to one of three commercially available study diets utilizing common protein sources (Purina Pro Plan Savor lamb, Purina Pro Plan Sport chicken, Purina Pro Plan Focus salmon) for a minimum of 30 days prior to initiation of the study. Following acclimation, dogs in the Test group began a daily regimen of oral probiotic (Fortiflora® Purina, St. Louis, MO) top-dressed on their midday feeding. Control dogs received their midday feeding with no probiotic. All dogs were sampled once weekly via oral pediatric swabs across the 7-week study. Next generation sequencing (Illumina, MiSeq) was utilized to develop microbial profiles specific to treatment, diet, and time. Bacterial composition was dominated by eight phyla (Proteobacteria 43.8%, Bacteroidetes 22.5%, Firmicutes 18.9%, Actinobacteria 6.1%, Fusobacteria 3.6%, Gracilibacteria 2.1%, SR1 Absconditabacteria 1.5%, and Saccharibacteria 1.3%) representing more than 99% of the relative abundance of the microbial composition. Probiotic administration failed to impact relative abundance at any taxonomic level (*P* > 0.05). Similarly, no effect on the oral microbiota was measured for diet (*P* > 0.05). Comparison using a Jaccard Index demonstrate a consistent microbial profile over the 7-week study with no impact evidenced by study week (*P* = 0.19). The data also revealed a profile of ubiquitous taxa that were present across all dogs and all samples regardless of breed, sex, diet, treatment or other factors. These genera include *Actinomyces, Corynebacterium, Capnocytophaga, Flavobacterium, Gemella, Abiotrophia, Streptococcus, and Frederiksenia*. These data demonstrate the stability of canine oral microbiota over time.

## Introduction

The microflora of the oral cavity is highly diverse and has been implicated in serious disease processes (1). In humans, the mouth of the average adult hosts an oral microbiome consisting of 50–100 million bacteria further characterized into about 200 different species (2). Recent work in dogs has shown that the canine oral microbiota harbors similar numbers, but the population varies significantly from that of humans (3). Prior to the completion of that study, the canine oral microbiome had only been characterized utilizing culture methods but has now been successfully studied utilizing modern 16s rRNA sequence analysis, allowing for a more comprehensive understanding of the oral microbiota as a whole (3).

Many factors affecting the oral microbiota including food intake, environmental conditions, health status, and age of the host have been described (4, 5). When the flora is stable and healthy, it serves as a protective barrier, but when one or more of these factors are altered, the bacteria existing synergistically with its host can shift in composition, resulting in opportunistic infection (2). The oral microbiota of working dogs may vary by the assigned job, according to a prior study utilizing detection dogs. The authors reported a significant difference in bacterial community composition within the oral cavity based on job assignment (5).

A wide range of diseases including periodontitis, dental caries, and endodontic disease have been associated with changes in the oral microbiome (2). Other work has shown that oral microbiota can serve as a biomarker for cardiac disease, bacterial pneumonia, and pancreatic cancer (2). In order to determine the role of the canine oral microbiota in infection and disease, the oral microbiota of healthy dogs must be further characterized. Longitudinal studies in humans indicate that while the gut microbiota changes over time (7), the oral microbiota are less dynamic and appear to demonstrate greater stability (8).

Probiotics are living microorganisms that when administered at appropriate doses, provide a health benefit to the host (6). In a recent review paper, Bustamante evaluated several studies focused on the utilization of probiotics as a treatment for various dental diseases in humans, including halitosis, dental caries, and periodontitis. The results of this meta-analysis indicated that there may be some benefit to using probiotics to treat halitosis and periodontitis. However, the studies evaluated varied greatly in design and population size, making them difficult to directly compare results. The effect of probiotics on oral health has not been evaluated in canines.

In some cases, oral bacteria have been demonstrated to seed the intestine and cause disease. Olsen et al. discussed that large amounts of oral bacteria are translocated to the gut on a regular basis, but are generally poor colonizers in the intestines as the bacteria must survive the low pH of the stomach. However, in certain disease states, it is possible for the bacteria of the oral cavity to translocate and cause intestinal disease (9). The link between oral and gut microbiota health is important and further research is needed to develop a more robust understanding of the connection between resident microbial colonies within these two distinct gastrointestinal locations.

This study was designed to characterize the canine oral microbiota in dogs receiving a daily oral probiotic and to assess the stability of the microbiome over time. We hypothesized that administration of an oral probiotic would impact the oral taxa in treated dogs. Additionally, authors predicted that time would affect the bacterial community present within the oral cavity of all dogs.

## Materials and Methods

### Animals and Diets

Procedures for this work were approved in advance by the Institutional Animal Care Use Committee at the University of Pennsylvania (protocol # 806541). Study participants included an experimental group of 14 dogs in training at the Penn Vet Working Dog Center (PVWDC). One subject was removed from the study due to inability to comply with the restrictive diet required for study inclusion. Each dog underwent a comprehensive examination by a licensed veterinarian prior to inclusion in the study and were determined to be healthy, with no observable medical or dental abnormalities, making them eligible for participation. Due to the possible effect on oral microbiota, no dental cleanings were performed over the course of the study. Participants included German shepherd dogs (*n* = 3), Dutch shepherds (*n* = 5), Belgian Malinois (*n* = 1), and Labrador retrievers (*n* = 4). Dogs were an average of 13.5 months old, weighing an average of 26 kg (see Table 1). Exclusion criteria included prior antibiotic or probiotic administration within 30 days of study initiation.

**Table 1:** Characteristics (breed, age, diet consumed, weight, and treatment group) of study participants enrolled in 7-week study.

**Dog**	**Breed**	**Age (months)**	**Diet**	**Weight (kg)**	**Treatment Group**
Cody	German Shepherd	15	Lamb	29.3	Control
Déjà vu	German Shepherd	19	Salmon	24.0	Control
Tallon	Labrador retriever	10	Chicken	37.0	Control
Joey	Dutch Shepherd	14	Lamb	24.2	Control
Ellie	German Shepherd	16	Lamb	22.9	Control
Tanner	Labrador retriever	10	Salmon	33.3	Control
Lucy	Dutch Shepherd	14	Chicken	23.2	Control
Callie	Dutch Shepherd	14	Salmon	22.5	Test
Jolie	Dutch Shepherd	14	Lamb	21.6	Test
Murphy	Dutch Shepherd	14	Chicken	27.7	Test
Blitz	Belgian Malinois	10	Lamb	22.6	Test
Jenner	Labrador retriever	13	Chicken	28.7	Test
Willow	Labrador retriever	13	Chicken	25.2	Test

Study participants were acclimated to commercially available assigned diets (Nestlé Purina PetCare, St. Louis, MO) consisting primarily of chicken, lamb, or salmon (Table 2) for a minimum of 4 weeks prior to initiation of the study. Block randomization was utilized to sort the dogs into either a test (*n* = 6) or control (*n* = 7) group. Study participants were blocked by breed, age, sex and diet. Study diets (Table 2) were maintained throughout the 7-week study with no novel food items introduced. Foster families, who cared for study participants overnight, were instructed prior to study start that no novel foods were to be introduced during the 7-week study period. Understanding of this requirement was confirmed by individual meetings with each foster family.

**Table 2:** Nutritional Content and primary ingredients for the three diets utilized throughout the 7 week study period.

**Diet**	**Protein Source**	**Protein (%) min**	**Fat (%) min**	**Fiber (%) max**	**Kcal/cup**	**Main Ingredients**
Purina Pro Plan Savor	Lamb	26	16	3	389	Lamb, rice flour, corn gluten meal, whole grain wheat, chicken by-product meal
Purina Pro Plan Sport	Chicken	30	20	3	475	Chicken, corn gluten meal, brewer's rice, animal fat, poultry by-product meal
Purina Pro Plan Focus	Salmon	26	16	4	429	Salmon, barley, ground rice, canola meal, oat meal

Dogs were individually housed in kennels while at the PVWDC during the day for 5 days a week, and in foster homes in the evenings and on weekends. Study participants in the Test group received a single packet of oral probiotic (Fortiflora®, Nestle Purina PetCare, St. Louis, MO) once daily during their midday meal during weeks 1–4 of the 7-week trial. The probiotic was in powder form and one packet of powder was sprinkled on top of dry kibble of each midday meal. Study participants in the Control group received no oral probiotic but maintained the same feeding regimen. All dogs were maintained on an identical parasite control regimen utilizing ivermectin/pyrantel (Heartgard®, Merial, Duluth, Gerorgia) and afoxolaner (Nexgard®, Merial, Duluth, Georgia) according to weight.

### Sample Collection and Analysis

Oral samples were collected once weekly by a single trained technician, over the course of the 7-week study period, with food, water, and toys withheld for 30 min prior to collection. Saliva was collected via oral pediatric swab (SalivaBio, State College, PA) introduced on the left side of the mouth between the cheek and gum with gentle massage for 60–90 s. Saturated swabs were immediately placed on ice and frozen (−80°C) within 60 min of collection. Samples were shipped overnight to Southern Illinois University for further processing. Upon receipt, swabs were thawed at room temperature for 30 min and centrifuged for 5 min at 1,500 X g (PowerSpin LXTM) to separate the saliva for quantification. A sample preparation containing 50 μl of saliva and 1.5 cm of oral swab was processed for DNA extraction according to manufacturer's guidelines (Zymo®) and eluted with 10 μl of DNA free water. Extracted DNA was assessed for purity (260/280 nm wavelength) and concentration (ng/ul) using a nanophotometer (Implen, Inc. NanoPhotometer™ P330, Westlake Village, CA). Quantified samples were stored (−80°C) prior to overnight shipment to Nestlé Purina for next generation sequencing.

Libraries were prepared using 1 μl quantified by Quant-It Pico Green (Fisher # P7589) on a Bio Tek Flx800 fluorometer, and 20 μl of a 1:10 dilution of each sample was run on a 1% Agarose E-Gel (Fisher # G7008-01) along with a 15 Kb high range ladder (Fisher # 12-352-019) to verify DNA integrity. Samples were then normalized to 5 ng/μl using 10 mM Tris HCl, pH = 8.5 (Tris HCl- Bioworld # 42020414-1) and once again quantified by Pico Green. 2.5 μl of the 5 ng/μl DNA was added to 22.5 μl of a master mix containing 12.5 μl 2X KAPA HiFi HotStart Ready Mix (Fisher # NC029523), and 5 μl each of Amplicon Forward and Reverse Primers (1 μM each), which span the V_3_-V_4_ region of the 16S rRNA (See Supplementary Information for sequences) as previously described (10). Plates were covered, vortexed and spun down. PCR was performed in a G-Storm GS-4 Thermal Cycler as follows: 95°C for 3 min, 25 cycles of 95°C for 30 s, 55°C for 30 s, 72°C for 30 s, and a final extension of 72°C for 5 min, then a hold at 4°C. One microliter of each PCR product was checked by Pico Green to confirm amplification and 24 samples were picked to have 1 μl run on a Bioanalyzer DNA 1000 Chip (Agilent # 5067-1504). The expected size for amplicons is around 570 bp. PCR products were cleaned using the AMPure XP Beads (Fisher # NC9933872), where 20 μl of AMPure XP beads were added to each PCR Product and mixed by pipetting up and down 10 times. Samples were incubated at room temp for 5 min without shaking. The plates were placed on a magnetic bead stand for 3 min to allow the supernatant to clear. The supernatants were removed and discarded, and the samples were washed twice with 200 μl of 80% Ethanol (Ethanol – 200Proof – Greenfield Global USA # 111000200CSPP), with wash supernatants being discarded. The plates were then air dried for 10 min to allow any remaining ethanol to evaporate. Plates were removed from the magnetic stand and 55 μl of Tris HCl was added to each sample. Plates were gently vortexed to resuspend the bead pellet and release the DNA from the beads. After a quick spin down, the plates were incubated at room temperature for 2 min. The plates were placed back on the magnetic stand for 3 min, then 50 μl of the cleared supernatant was transferred to a clean PCR plate.

A second PCR (Index PCR) was run on each of the 1st PCR products as follows: 5 μl of the 1st PCR product was added to 35 μl of a master mix containing 25 μl 2X KAPA HiFi HotStart Ready Mix, and 10 μl of Molecular grade water (Fisher # BP2819-1). Five microliter of Nextera XT Index Primer 1 (N7xx) and 5 μl of Nextera XT Index Primer 2 (S5xx) (Illumina # FC-131-200X), where X is #1–4 for sets A, B, C or D, (See Supplementary Information for sequences) were added to the samples, so that each sample had a different and unique combination of the two primers. Plates were covered, vortexed and spun down. PCR was performed as follows: 95°C for 3 min, 8 cycles of 95°C for 30 s, 55°C for 30 s, 72°C for 30 s, and a final extension of 72°C for 5 min, then a hold at 4°C. The Index PCR products were cleaned as above with the following differences: 56 μl of AMPure XT beads are added to each well, 30 μl Tris HCl was used to resuspend beads and release the DNA, and finally 25 μl of cleared supernatant was transferred to a clean PCR plate. One microliter of each PCR Product was quantified by Pico Green and 24 samples were selected to have 1 μl run on a Bioanalyzer DNA 1000 Chip. The expected size for the Index PCR Amplicons was 630–650 bp. The values from the Pico Green were used to calculate the concentration in nM using the below formula where 630 bp is the average library size:

(1) $$concentration\;in\;nM=\frac{\text{concentration in ng/ul}}{\text{660 }\frac{\text{g}}{\text{mol}}\text{x average library size x }{\text{10}}^{\text{6}}}$$

Each sample was normalized to 8 nM (due to low concentrations of 2nd PCR Product) using Tris HCl and the concentration checked by Pico Green. Equal amounts of each sample (3 μl) were combined in one tube to give a 8 nM pool. The 8 nM pool was quantified by KAPA ROX LOW QPCR kit (Kapa Biosystems KK4873, Fisher Scientific #NC833039) on an ABI 7500 Fast qPCR system as follows: In duplicate - 2 μl of the library was diluted to 1:1,000 using Tris HCl with 0.05% Tween 20 (Sigma P1379-100ML), then serial diluted to 1:8,000. Sixteen microliter of a master mix containing 12μl 2X SYBR Fast with Primer Premix (From KAPA Kit) and 4 μl molecular grade water was added to each well of a qPCR plate. Four microliter (in triplicate) was added for each of the 6 standards from the kit as well as from each dilution and run using the following program: denature at 95°C for 5 min, 35 cycles of 95°C for 30 s, 60°C for 45 s. After the run, a melt curve analysis was performed on the samples with expected melt temperature around 85°C. Using the concentration from the qPCR, the pool was diluted down to 4 nm with Tris HCl and a KAPA qPCR was repeated, using 1:500 and 1:1,000 dilutions on six different 4 nM samples. Pool concentration was diluted and checked until confirmed at 4 nm±.

A MiSeq cartridge was thawed in room temperature water then placed at 4°C until needed, while the vial of HT-1 was thawed at room temperature then placed on ice. A sample sheet was made containing the samples and their respective index pairs using Illumina Experiment Manager and transferred to the MiSeq before loading the cartridge. Two microliter of 10 nM PhiX control (Illumina # FC-110-3001) was diluted to 4 nM with 3 μl Tris HCL with 0.1% Tween 20. Next, 5 μl 0.2 N NaOH (1N - VWR # BJ65982-1P) was added to 5 μl of the 4 nM DNA Library and 5 μl of the 4 nM PhiX individually, and incubated for 5 min at room temperature to denature the DNA into single strands. The denatured DNA and PhiX were diluted to 20 pM using 990 μl ice cold HT-1. Both the 20 nM DNA and 20 nM PhiX were diluted again to final loading concentration of 11pM (330 μl Library/PhiX plus 270 μl Ice Cold HT-1). Finally, 504 μl of the library and 96 μl of the PhiX (both at 11 pM) were combined. Six-hundred microliter of the Library/PhiX mix was loaded into a MiSeq Reagent Kit v2 (500cycle) (Illumina # MS-102-2003), placed into the Illumina MiSeq along with a fresh Flow Cell, and run for 250 x 2 cycles. Results from the Illumina Sequencing Analysis Viewer showed a loading density of 522 K/mm^2^ and a 23.87% PhiX alignment with 9.43 M Reads passing filter.

### Sequencing

A total of 17,386,296 reads were obtained from the 91 samples with an average sequencing depth of 88,706 reads. Paired-end reads were merged and assembled into a single read using the software PEAR with default settings (11). The average rate of assembly was 95.4%. Sequences with <350 bases or >475 bases were removed. All sequences from different samples were combined to create a single file and redundant sequences were removed to create unique sequences. The de-replicated sequences were sorted and clustered into operational taxonomic units (OTUs) based on minimal 97% identity using the UPARSE-OTU clustering algorithm (12, 13). Chimeric sequences were detected and discarded. The OTU table was built from 1,839 OTUs. Taxonomy assignment was performed using the kmer-based K-nearest neighbor search algorithm implemented in Mothur (version 1.39.5) (13) by searching the reference sequence file from the SILVA database (release 128) (14). Sequence alignment was performed using PyNAST (15). A phylogenetic tree was built from the aligned sequences using FastTree (16, 17). QIIME (version 1.9.1) wrapper functions were called for making taxonomy assignment, OTU table and phylogenetic tree (18).

Data analysis was performed using R version 3.5.2 (19) utilizing the package phyloseq (20) version 1.26.1, to import data and calculate alpha and beta diversity, unless otherwise noted. Samples containing <3,000 reads were discarded, leaving 88 oral samples for analysis. For relative abundance calculations, absolute abundance was converted into relative abundance, and OTUs where the maximum in any sample was <0.1% were removed. Plots were generated using ggplot2 version 3.1.1 (21). Outcomes of interest were differences related to probiotic supplementation and study period. Beta diversity analyses include both Bray Curtis and Unifrac (weighted and unweighted) measures represented as principal coordinates analysis (PCoA) plots, with statistically significant differences determined by PERMANOVA. Alpha diversity analysis included Simpson, invSimpson, Fisher, Chao1, Shannon, and Observed. Significance was established at *P* ≤ 0.05. To examine the differences in relative abundance between bacteria, linear mixed effects models were used to account for the fact that the dogs were measured at multiple times. Mixed models were run in R using the lme4 package version 1.1-21 (22). Dog name was entered as a random effect where the intercept was allowed to vary between dogs. *P*-values were obtained using the Satterthwaite approximation of degrees of freedom. Probiotic supplementation, diet, and study period were entered as fixed effects. Resulting *P*-values from the linear mixed models were adjusted for multiple comparisons using the Benjamini–Hochberg false discovery rate. If the resulting Q-value was deemed significant (Q ≤ 0.05), *post-hoc* comparisons were made between groups.

## Results

### Microbial Composition

Oral bacterial DNA were harvested to generate 1,609,186 sequences with a mean (±SD) of 17,683 (±5,466) sequences per sample. To provide adequate depth, samples with fewer than 3,000 reads were discarded, maintaining 88 of the 91 samples for microbiota analysis. Next generation sequencing identified 155 individual taxa collected via non-invasive saliva sampling during the 7 week study. The full taxonomy (using a provisional six level) is presented in Supplementary Table 1. Four phyla contained >80% of all taxa identified. The phylum Firmicutes was represented by 46 individual taxa and was dominated by taxa from the class Bacilli (*n* = 20) and Clostridia (*n* = 20). Proteobacteria contained 38 individual taxa, 11 of which are currently undescribed in bacterial databases. Bacteroidetes contained 28 individual taxa, including four taxa from the Genus *Capnocytophaga*. Fifteen Actinobacteria were identified. Identified taxa were from the genera *Actinomyces, Corynebacterium, Leucobacter, Arthrobacter, Reothia, Propionibacterium and Propioniciclava*. In addition, taxa from 22 classes, 34 orders, 52 families, and 92 genera were present. Interestingly, the data also revealed a profile of ubiquitous (core) taxa that were present across all dogs at every time point. These taxa appear to be impervious to breed, sex, diet, treatment or other factors. These resilient genera *include Actinomyces, Corynebacterium, Capnocytophaga, Flavobacterium, Gemella, Abiotrophia, Streptococcus*, and *Frederiksenia*.

### Probiotic Administration and Diet

Oral administration of the probiotic yielded no impact on relative abundance (*P* > 0.05) when test and control groups were compared (Table 3). Relative abundance of the oral microbiota was unaffected by diet type with no change measured for dogs on chicken, lamb or salmon formulas (*P* > 0.05) as shown in Supplementary Table 2. Diet and probiotic administration did not affect alpha diversity values (*P* > 0.05 for all metrics measured). Furthermore, UniFrac values were also unaffected by diet for both unweighted (*P* = 0.408) and weighted measures (*P* = 0.503) of diversity. Similarly, probiotic administration failed to influence the oral microbiota with weighted (*P* = 0.889) and unweighted (*P* = 0.947) values remaining unaffected.

**Table 3:** Relative abundance (%) of predominant oral microbial taxa present in canines administered oral probiotic (test) vs. control during seven weeks of repeated salivary sampling.

**Treatment Group (±SD)**			
**Phylum**	**Control**	**Test**	***P*****-value**
Proteobacteria	43.14 (22.86)	44.58 (19.95)	0.8823
Bacteroidetes	20.55 (16.39)	24.72 (16.44)	0.5469
Firmicutes	22.44 (17.49)	14.75 (7.99)	0.3327
Actinobacteria	5.98 (5.33)	6.3 (7.26)	0.8823
Fusobacteria	3.57 (4.00)	3.72 (4.44)	0.8823
Gracilibacteria	1.58 (1.37)	2.57 (2.96)	0.2836
Saccharibacteria	1.15 (1.79)	1.45 (1.32)	0.5469
SR1 (Absconditabacteria)	1.18 (1.24)	1.8 (1.69)	0.2836
**Class**			
Gammaproteobacteria	35.93 (25.87)	35.76 (23.85)	0.9139
Flavobacteriia	15.63 (13.25)	21.08 (14.41)	0.5255
Bacilli	16.51 (17.94)	9.99 (7.57)	0.5837
Betaproteobacteria	6.94 (5.65)	8.61 (5.20)	0.5909
Actinobacteria	5.35 (4.84)	5.56 (6.65)	0.9038
Clostridia	5.36 (4.80)	4.26 (3.84)	0.7120
Bacteroidia	4.92 (7.28)	3.63 (4.71)	0.5909
Fusobacteriia	3.57 (4.00)	3.72 (4.40)	0.9038
**Order**			
Pseudomonadales	21.72 (30.62)	22.72 (28.73)	0.9149
Flavobacteriales	15.63 (14.41)	21.08 (3.84)	0.6648
Lactobacillales	12.68 (13.89)	8.02 (5.66)	0.6648
Pasteurellales	10.54 (12.62)	8.7 (7.52)	0.7109
Clostridiales	5.36 (4.80)	4.26 (3.84)	0.7565
Burkholderiales	4.49 (4.56)	5.1 (3.66)	0.8681
Bacteroidales	4.92 (7.28)	3.63 (4.71)	0.6648
Fusobacteriales	3.57 (4.00)	3.72 (4.44)	0.9149
Bacillales	3.83 (9.80)	1.97 (3.70)	0.6648
Neisseriales	2.45 (2.45)	3.52 (2.98)	0.6527
Actinomycetales	2.48 (3.48)	2.76 (4.23)	0.8734
Xanthonomonadales	1.81 (2.05)	2.4 (2.04)	0.6648
Cardiobacteriales	1.67 (1.88)	1.94 (1.50)	0.7847
Micrococcales	1.61 (2.15)	1.36 (2.04)	0.8734
Gracilibacteria bacterium canine oral taxon 394	0.95 (0.94)	1.57 (1.86)	0.6527
**Family**			
Moraxellaxceae	20.22 (28.82)	21.7 (26.88)	0.9537
Flavobacteriaceae	15.63 (13.25)	21.08 (14.41)	0.6700
Pasteurellaceae	10.54 (12.62)	8.7 (7.52)	0.7411
Streptococcaceae	7.65 (8.53)	4.26 (3.22)	0.6700
Aerococcaceae	4.48 (6.59)	3.38 (3.18)	0.7411
Porphyromonadaceae	4.22 (6.00)	3.03 (3.91)	0.7239
Neisseriaceae	2.45 (2.45)	3.52 (2.98)	0.6094
Burkholderiaceae	2.68 (3.11)	2.64 (2.27)	0.9682
Fusobacteriaceae	2.38 (2.95)	3.37 (4.22)	0.7239
Actinomycetaceae	2.48 (3.48)	2.76 (4.23)	0.9089
Bacillales Family XI	2.05 (3.31)	0.99 (1.59)	0.6094
Comamonadaceae	1.81 (2.05)	2.45 (1.91)	0.7239
Xanthomonadeaceae	1.81 (2.05)	2.4 (2.04)	0.7239
Cardiobacteriaceae	1.67 (1.88)	1.94 (1.50)	0.8422
Peptostreptococceceae	1.62 (1.78)	1.13 (1.14)	0.7411
Defluviitaleaceae	1.52 (1.55)	1.74 (1.95)	0.8707
Pseudomonadaceae	1.49 (10.17)	1.02 (6.53)	0.9091
Planococcaceae	1.46 (9.15)	0.89 (3.48)	0.9020
Clostridiales Family XII	1.11 (1.33)	0.9 (0.47)	0.6474
Gracilibacteria bacterium canine oral taxon 394	0.95 (0.94)	1.57 (1.86)	0.6094
**Genus**	
*Psychrobacter*	13.63 (28.93)	15.48 (29.34)	0.8905
*Bergeyella*	8.27 (8.45)	10.11 (8.19)	0.6766
*Streptococcus*	7.65 (8.53)	4.26 (3.22)	0.6714
*Pasteurellaceae uncultured*	6.46 (11.78)	3.09 (4.82)	0.6714
*Moraxella*	5.15 (5.81)	5.8 (5.62)	0.7859
*Capnocytophaga*	4.07 (4.48)	5.47 (3.75)	0.6714
*Porphyromonas*	3.91 (5.60)	2.77 (3.72)	0.6714
*Abiotrophia*	3.75 (6.54)	2.05 (2.85)	0.2862
*Flavobacterium*	3.3 (5.72)	5.5 (6.43)	0.1998
*Frederiksenia*	2.95 (3.23)	3.72 (2.77)	0.6714
*Lautropia*	2.68 (3.11)	2.64 (2.27)	0.9792
*Actinomyces*	2.48 (3.48)	2.76 (4.23)	0.8446
*Fusobacterium*	2.38 (2.95)	3.37 (4.22)	0.6714
*Gemella*	2.05 (3.31)	0.99 (1.59)	0.0885
*Xanthmonadeaceae-Uncultured genus*	1.66 (1.89)	2.39 (2.04)	0.6714
*Difluviitaleaceae UGC-011*	1.52 (1.55)	1.74 (1.95)	0.7901
*Pseudomonas*	1.49 (10.17)	1.02 (6.53)	0.8905
*Cardiobacteriaceae ambiguous taxa*	1.47 (1.60)	1.7 (1.49)	0.6113
*Neisseria*	1.32 (1.35)	1.96 (1.60)	0.6714
*Corticibacter*	1.31 (1.62)	1.71 (1.58)	0.7131
*Absconditabacteria ambiguous taxa*	1.18 (1.24)	1.8 (1.69)	0.6714
*Fusibacter*	1.11 (1.33)	0.90 (0.97)	0.7859
*Gracilibacteria bacterium canine oral taxon 394*	0.95 (0.94)	1.57 (1.86)	0.6714
*Aerococcoceae NA*	0.73 (1.01)	1.33 (1.92)	0.6714

### Time Elapsed

**Figure 1:**
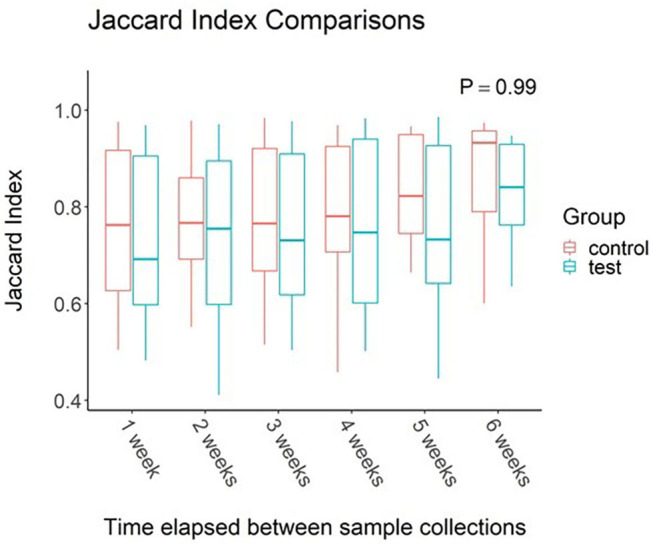
Impact of time elapsed on salivary microbiota for dogs administered oral probiotic vs. control. Jaccard index values were calculated between samples for each dog. Values were then grouped based on weeks elapsed between sample collections. Boxplots represent the distribution of Jaccard Index values when said number of weeks had elapsed between two samples. Upper and lower hinges of boxplots represent first and third quartiles. Upper whiskers extend from hinge to the largest value no further than 1.5 * IQR (inter-quartile range) of the hinge. Lower whiskers extend from the hinge to the smallest value at most 1.5 * IQR of the hinge. Middle line represents the median value.

When effects associated with time were considered, the oral microbiota appear to be unaffected. Study week did not have an effect on alpha diversity values (*P* > 0.05 for all metrics measured). Jaccard Index (Figure 1) comparisons between elapsed time reveal no change between samples collected across the 7-week study regardless of time elapsed between samplings (*P* = 0.21).

## Discussion

The predominant phyla within the test subjects were Proteobacteria, Bacteroidetes, Firmicutes, Actinobacteria, Fusobacteria, Gracilibacteria, SR1 Absconditabacteria, and Saccharibacteria, representing more than 99% of the relative abundance of the microbial composition. Although the taxa represented in this dataset are consistent with findings in previous literature, the relative abundance reported appears to vary (4, 5, 24). It is likely that the variations in relative abundance are associated with differences in extraction kit, collection tool, and collection technique (23). In addition to differences in extraction and collection methods, it is likely that there is natural variation present within different populations of dogs living in different environments and geographical locations. The dogs utilized in the current study lived in a more diverse environment than dog's purpose bred for research. This population was also made up of dogs bred from working lines. Comparable studies have utilized dogs from client-owned populations, research, as well as working dog populations (5, 27, 30).

The human oral microbiome has been well characterized, however the canine oral microbiome has not (3, 4, 25). It has also been shown that the bacterial presence within the canine oral cavity is dissimilar to that of the human oral cavity, with the microbial composition only 28% similar in one study (25) and 16.4% similar in another (3).

Factors contributing to fluctuations in the gastrointestinal microbiota have been well characterized across multiple species, however, many gaps remain in our understanding of the canine oral microbiota (4, 9, 23, 26). The consistency in relative abundance shown in the current study demonstrates remarkable stability within the oral microbial ecosystem when faced with varied dietary protein sources, breed, sex and time. These findings are consistent with findings documented in humans, when stability of the gut microbiome and the oral microbiome were compared following oral antibiotic administration (8). In one study examining impacts on both oral and gastrointestinal microbiota following antibiotic administration, authors reported stability within the oral microbial profile, despite considerable changes within the gut microbiota that included reduced diversity for several months post-antibiotic administration (8). Ciprofloxacin, clindamycin, amoxicillin, and minocycline were selected for this study because each represent a different antibiotic class with different mechanisms of action, but all are widely prescribed (8). Other work evaluating the impact of dental cleanings on the oral microbiota demonstrated that although dental prophylaxis did result in changes in relative abundance, the microbial community quickly reverted back to its original composition, indicating that the oral microbiota is stable, consistent, and resilient (27).

The current study also describes ubiquitous taxa present in samples across all test subjects, suggesting that the oral microbiota may be highly conserved across dogs in general, as these taxa were unaffected by diet, probiotic administration, or time. Other authors have theorized that selective constraint associated with the oral microbiota has produced an oral environment that allows the resident population to remain unaffected by competitive efforts of non-residents, despite the exposure of the oral cavity to the external environment and various dietary regimens (28). This is quite interesting because other studies have documented changes in the gut microbiota in the face of oral disease, specifically with the presence of *Porphymonas gingivalis* (9). The 13 dogs enrolled in the current study were young and free of evidence of oral disease. It is likely that when the health of the oral environment is compromised, opportunistic infection of the gut microbiota, as well as the development of systemic disease can occur (9, 29).

The oral microbiota was consistent over time as well. All test subjects spent significant amounts of time in varied environments as they all resided in different foster homes. However, oral microbial composition remained unchanged throughout the 7-week study period which further support the theory of stability and resistance to colonization within the oral cavity. It is possible that this is an evolutionary adaptation designed to protect the host from invasive taxa. Though there are limited studies evaluating changes in the canine oral microbiota over time, Flancman was able to demonstrate similar microbial resiliency when comparing oral microbiota samples pre and post dental prophylaxis in dogs (27). Other work in detection dogs examined buccal swabs collected at baseline and again 7 weeks later. When authors examined dogs housed together on similar diets, they found differences only due to age and breed, which are widely accepted factors known to impact microflora (5). Geographical location was also a factor for different microbial profile. However, dogs were from different facilities, of different breeds, and with different exercise programs so it may be that other factors contributed to the differences attributed to geographical location. Future studies utilizing controlled diets, similar genetics, age groups and exercise regimens are needed to identify specific impacts to canine oral salivary microflora. Additionally, studies have shown that methodology can significantly impact sequencing results (32). The prior study measuring oral microbiota across two time points utilized buccal swabs and different extraction techniques (5). It is possible that some differences may be attributed to those slight changes in laboratory procedures.

The secondary hypothesis of this study that probiotic administration would alter the composition of oral flora was unsupported. This is in contrast to prior work evaluating the effect of a probiotic containing *Streptococcus thermophilus* SP4, *Lactobacillus plantarum* 14D, and *Lactobacillus rhamnosus* SP1 supplement in cats and dogs. This supplement was administered in the same manner as the current study, as a powder on top of dry feed. The authors found that the overall relative abundance of infectious microbes was reduced (30). The authors selected those particular bacteria due to their ability to survive in the oral cavity (30). Different results may be due to selection based on natural resident taxa with an ability to colonize as well as taxa identified as beneficial. Non-specific probiotics may have a positive, negative, or neutral effect on other microbial communities within the body (31). It is likely that the typical probiotic microorganisms utilized in our study were not well-suited to residency within the oral environment as they lack binding capabilities appropriate to that environment.

This study had limitations in that a low number of test subjects were enrolled. Study numbers were limited by current enrollment in a training program for working canines. Training program candidates were excluded as a result of medical treatment with antibiotics and probiotics, further reducing the number of subjects available. Although it may be considered a limitation, dogs enrolled in the study lived in a more varied environment than kennel-housed animals in highly controlled settings. They consumed one of three diets and were not all eating the same food, though the diet for each individual dog did not change throughout the course of the study. Though every effort was made to control confounding variables, an individual home-living environment setting inevitably results in greater environmental variability than a group-housed kennel environment. Even with the natural variation of individual homes, the oral microbiota in these dogs remained stable over time. Another limitation of this study is that control dogs were not given a placebo powder during the probiotic administration phase. However, dog handlers and sample collectors were blinded to the group each participant was assigned to and did not participate in feeding. Future studies are needed to further understand and define the relationship between the oral microbiota and the gut microbiota as well as the role of oral probiotics in the oral, gastrointestinal, and microbial health of the dog.

## Data Availability Statement

The datasets presented in this study can be found in online repositories. The names of the repository/repositories and accession number(s) can be found below: https://www.ncbi.nlm.nih.gov/, PRJNA645190.

## Ethics Statement

Procedures for this work were approved in advance by the Institutional Animal Care Use Committee at the University of Pennsylvania (protocol #806541).

## Author Contributions

SB participated in development of study design, study execution, data collection, manuscript writing and review. AN participated in data analysis and interpretation, development of figures and tables, and manuscript development. BZ participated in development of study design, and manuscript review. CO participated in development of study design, study coordination, and manuscript review. EP participated in development of study design, sample processing, data analysis, manuscript writing, and review. All authors read and approved the final manuscript.

## Conflict of Interest

The authors declare that the research was conducted in the absence of any commercial or financial relationships that could be construed as a potential conflict of interest.
