# Supplementary material for: An Assessment of the Stability of the Canine Oral Microbiota After Probiotic Administration in Healthy Dogs Over Time
Source: Front Vet Sci. 2020 Sep 11;7:616. doi: 10.3389/fvets.2020.00616 (PMC7517700; doi:10.3389/fvets.2020.00616)
Supplement: Supplementary file 1 [file Data_Sheet_1.docx]

**Supplemental Information**

16S Amplicon PCR Forward Primer = 5'

TCGTCGGCAGCGTCAGATGTGTATAAGAGACAGCCTACGGGNGGCWGCAG

16S Amplicon PCR Reverse Primer = 5'

GTCTCGTGGGCTCGGAGATGTGTATAAGAGACAGGACTACHVGGGTATCTAATCC

Forward overhang: 5’ TCGTCGGCAGCGTCAGATGTGTATAAGAGACAG‐[locusspecific

sequence]

Reverse overhang: 5’ GTCTCGTGGGCTCGGAGATGTGTATAAGAGACAG‐[locusspecific

sequence]

Nextera Transposase Adapters

Read 1

5’ TCGTCGGCAGCGTCAGATGTGTATAAGAGACAG

Read 2

5’ GTCTCGTGGGCTCGGAGATGTGTATAAGAGACAG

Nextera Index Kit – PCR Primers

Index 1 Read

5’ CAAGCAGAAGACGGCATACGAGAT[i7]GTCTCGTGGGCTCGG

Index 2 Read

5’ AATGATACGGCGACCACCGAGATCTACAC[i5]TCGTCGGCAGCGTC

Nextera XT Index Kit A S502, S503, S505 to S508, S510 AND S 511

N701 to N707, N710 to N712, N714 and N715

Nextera XT Index Kit B S502, S503, S505 to S508, S510 AND S 511

N716, N718 to N724 and N726 to N729

Nextera XT Index Kit C S513, S515 to S518 and S520 to S522

N701 to N707, N710 to N712, N714 and N715

Nextera XT Index Kit D S513, S515 to S518 and S520 to S522

N716, N718 to N724 and N726 to N729

Bases in Adapter i7 Index Name i7 Bases for Entry on Sample Sheet

TCGCCTTA N701 TAAGGCGA

CTAGTACG N702 CGTACTAG

TTCTGCCT N703 AGGCAGAA

GCTCAGGA N704 TCCTGAGC

AGGAGTCC N705 GGACTCCT

CATGCCTA N706 TAGGCATG

GTAGAGAG N707 CTCTCTAC

CAGCCTCG N710 CGAGGCTG

TGCCTCTT N711 AAGAGGCA

TCCTCTAC N712 GTAGAGGA

TCATGAGC N714 GCTCATGA

CCTGAGAT N715 ATCTCAGG

TAGCGAGT N716 ACTCGCTA

GTAGCTCC N718 GGAGCTAC

TACTACGC N719 GCGTAGTA

AGGCTCCG N720 CGGAGCCT

GCAGCGTA N721 TACGCTGC

CTGCGCAT N722 ATGCGCAG

GAGCGCTA N723 TAGCGCTC

CGCTCAGT N724 ACTGAGCG

GTCTTAGG N726 CCTAAGAC

ACTGATCG N727 CGATCAGT

TAGCTGCA N728 TGCAGCTA

GACGTCGA N729 TCGACGTC

Bases in Adapter i5 Index Name i5 Bases for Sample Sheet MiSeq, HiSeq 2000/2500

CTCTCTAT S502 CTCTCTAT

TATCCTCT S503 TATCCTCT

GTAAGGAG S505 GTAAGGAG

ACTGCATA S506 ACTGCATA

AAGGAGTA S507 AAGGAGTA

CTAAGCCT S508 CTAAGCCT

CGTCTAAT S510 CGTCTAAT

TCTCTCCG S511 TCTCTCCG

TCGACTAG S513 TCGACTAG

TTCTAGCT S515 TTCTAGCT

CCTAGAGT S516 CCTAGAGT

GCGTAAGA S517 GCGTAAGA

CTATTAAG S518 CTATTAAG

AAGGCTAT S520 AAGGCTAT

GAGCCTTA S521 GAGCCTTA

TTATGCGA S522 TTATGCGA
