# Supplementary material for: An Assessment of the Stability of the Canine Oral Microbiota After Probiotic Administration in Healthy Dogs Over Time
Source: Front Vet Sci. 2020 Sep 11;7:616. doi: 10.3389/fvets.2020.00616 (PMC7517700; doi:10.3389/fvets.2020.00616)
Supplement: Supplementary file 2 [file Data_Sheet_2.docx]

Supplemental Table 1. Taxonomy of the canine oral microbiota (beginning with order), present in canines during seven weeks of repeated salivary sampling.

| **Order** | **Family** | **Genus** | **Species** | **OTU** |
| --- | --- | --- | --- | --- |
| Actinomycetales | Actinomycetaceae | *Actinomyces* | Ambiguous taxa | 71 |
| Actinomycetales | Actinomycetaceae | *Actinomyces* | Ambiguous taxa | 79 |
| Actinomycetales | Actinomycetaceae | *Actinomyces* | NA | 160 |
| Actinomycetales | Actinomycetaceae | *Actinomyces* | NA | 195 |
| Actinomycetales | Actinomycetaceae | *Actinomyces* | NA | 448 |
| Actinomycetales | Actinomycetaceae | *Actinomyces* | NA | 98 |
| Corynebacteriales | Cornybacteriaceae | *Corynebacterium* | Ambiguous taxa | 127 |
| Corynebacteriales | Cornybacteriaceae | *Corynebacterium* | Ambiguous taxa | 154 |
| Micrococcales | Microbacteriaceae | *Leucobacter* | Ambiguous taxa | 30 |
| Micrococcales | Microbacteriaceae | NA | NA | 171 |
| Micrococcales | Microbacteriaceae | *Arthrobacter* | NA | 167 |
| Micrococcales | Microbacteriaceae | *Rothia* | Ambiguous taxa | 67 |
| Propionibacteriales | Propionibacteriaceae | *Propionibacterium* | NA | 114 |
| Propionibacteriales | Propionibacteriaceae | *Propioniciclava* | NA | 170 |
| Propionibacteriales | Propionibacteriaceae | uncultured | NA | 104 |
| Euzebyales | Euzebyaceae | uncultured | Ambiguous taxa | 63 |
| Bacteroidales | Bacteroidaceae | *Bacteroides* | Ambiguous taxa | 113 |
| Bacteroidales | Bacteroidaceae | *Bacteroides* | *bacterium NLAE-zl-C391* | 215 |
| Bacteroidales | Bacteroidaceae | *Bacteroides* | NA | 66 |
| Bacteroidales | Porphyromonadaceae | NA | NA | 203 |
| Bacteroidales | Porphyromonadaceae | *Petrimonas* | NA | 274 |
| Bacteroidales | Porphyromonadaceae | *Porphyromonas* | Ambiguous taxa | 101 |
| Bacteroidales | Porphyromonadaceae | *Porphyromonas* | Ambiguous taxa | 163 |
| Bacteroidales | Porphyromonadaceae | *Porphyromonas* | Ambiguous taxa | 164 |
| Bacteroidales | Porphyromonadaceae | *Porphyromonas* | Ambiguous taxa | 180 |
| Bacteroidales | Porphyromonadaceae | *Porphyromonas* | Ambiguous taxa | 218 |
| Bacteroidales | Porphyromonadaceae | *Porphyromonas* | Ambiguous taxa | 47 |
| Bacteroidales | Porphyromonadaceae | *Porphyromonas* | NA | 97 |
| Bacteroidales | Porphyromonadaceae | *Porphyromonas* | *Porites australiensis* | 934 |
| Bacteroidales | Porphyromonadaceae | *Porphyromonas* | *Porphyromonas canoris* | 112 |
| Bacteroidales | Porphyromonadaceae | *Tanerella* | Ambiguous taxa | 161 |
| Bacteroidales | Porphyromonadaceae | uncultured | Ambiguous taxa | 173 |
| Bacteroidales | Prevotellaceae | *Alloprevotella* | Ambiguous taxa | 181 |
| Bacteroidales | Prevotellaceae | *Alloprevotella* | NA | 201 |
| Bacteroidales | Prevotellaceae | *Alloprevotella* | *Prevotella sp canine oral taxon 282* | 178 |
| Bacteroidales | Prevotellaceae | *Alloprevotella* | *Prevotella sp canine oral taxon 372* | 172 |
| Flavobacteriales | Flavobacteriaceae | *Bergeyella* | Ambiguous taxa | 3 |
| Flavobacteriales | Flavobacteriaceae | *Bergeyella* | Ambiguous taxa | 87 |
| Flavobacteriales | Flavobacteriaceae | *Capnocytophaga* | Ambiguous taxa | 74 |
| Flavobacteriales | Flavobacteriaceae | *Capnocytophaga* | *Capnocytophaga sp canine oral taxon 329* | 122 |
| Flavobacteriales | Flavobacteriaceae | *Capnocytophaga* | NA | 43 |
| Flavobacteriales | Flavobacteriaceae | *Capnocytophaga* | NA | 580 |
| Flavobacteriales | Flavobacteriaceae | *Flavobacterium* | NA | 8 |
| Flavobacteriales | Flavobacteriaceae | *Flavobacterium* | uncultured bacterium | 103 |
| Bacillales | Family XI | *Gemella* | Ambiguous taxa | 26 |
| Bacillales | Planococcaceae | *Psychrobacillus* | uncultured bacterium | 150 |
| Bacillales | Planococcaceae | *Sporosarcina* | NA | 123 |
| Bacillales | Planococcaceae | *Sporosarcina* | NA | 249 |
| Bacillales | Planococcaceae | *Sporosarcina* | uncultured bacterium | 86 |
| Bacillales | Staphylococcaceae | *Jeotgalicoccus* | uncultured bacterium | 265 |
| Bacillales | Staphylococcaceae | *Macrococcus* | Ambiguous taxa | 227 |
| Bacillales | Staphylococcaceae | *Staphylococcus* | Ambiguous taxa | 118 |
| Bacillales | Staphylococcaceae | *Staphylococcus* | NA | 243 |
| Bacillales | Staphylococcaceae | *Staphylococcus* | NA | 685 |
| Lactobacillales | Aerococcaceae | *Abiotrophia* | Ambiguous taxa | 25 |
| Lactobacillales | Aerococcaceae | NA | NA | 42 |
| Lactobacillales | Carnobacteriaceae | *Granulicatella* | Ambiguous taxa | 65 |
| Lactobacillales | NA | NA | NA | 166 |
| Lactobacillales | Streptococcaceae | *Streptococcus* | Ambiguous taxa | 153 |
| Lactobacillales | Streptococcaceae | *Streptococcus* | NA | 209 |
| Lactobacillales | Streptococcaceae | *Streptococcus* | NA | 24 |
| Lactobacillales | Streptococcaceae | *Streptococcus* | NA | 84 |
| Lactobacillales | Streptococcaceae | *Streptococcus* | *Streptococcus salivarius subsp. Thermophilus* | 49 |
| Lactobacillales | Streptococcaceae | *Streptococcus* | *Streptococcus sp. Canine oral taxon 297* | 23 |
| Clostridiales | Christensenellaceae | *Christensenellaceae R-7 group* | NA | 208 |
| Clostridiales | Defluviitaleaceae | *Defluviitaleaceae UCG-011* | NA | 270 |
| Clostridiales | Defluviitaleaceae | *Defluviitaleaceae UCG-011* | NA | 45 |
| Clostridiales | Family XI | *Parvimonas* | Ambiguous taxa | 147 |
| Clostridiales | Family XII | *Fusibacter* | Ambiguous taxa | 169 |
| Clostridiales | Family XII | *Fusibacter* | Ambiguous taxa | 88 |
| Clostridiales | Family XII | *Fusibacter* | NA | 140 |
| Clostridiales | Family XII | *Fusibacter* | *Peptostreptococcaceae bacterium feline oral taxon 067* | 105 |
| Clostridiales | Lachnospiraceae | *Catonella* | Ambiguous taxa | 106 |
| Clostridiales | Lachnospiraceae | *Johnsonella* | *Lachnospiraceae bacterium canine oral taxon 399* | 210 |
| Clostridiales | Lachnospiraceae | *Johnsonella* | NA | 285 |
| Clostridiales | Peptoccoceae | *Peptococcus* | Ambiguous taxa | 133 |
| Clostridiales | Peptostreptococcaceae | *Eubacterium yuri group* | Ambiguous taxa | 102 |
| Clostridiales | Peptostreptococcaceae | *Filifactor* | Ambiguous taxa | 116 |
| Clostridiales | Peptostreptococcaceae | *Peptostreptococcus* | Ambiguous taxa | 165 |
| Clostridiales | Peptostreptococcaceae | *Proteocatella* | Ambiguous taxa | 83 |
| Clostridiales | Peptostreptococcaceae | uncultured | Ambiguous taxa | 177 |
| Clostridiales | Peptostreptococcaceae | uncultured | Ambiguous taxa | 85 |
| Clostridiales | Ruminococcaceae | *Fastidiosipila* | *Clostridiales bacterium canine oral taxon 216* | 90 |
| Clostridiales | Ruminococcaceae | *Fastidiosipila* | *Clostridiales bacterium canine oral taxon 261* | 188 |
| Erysipelotrichales | Erysipelotrichaceae | *Anaerorhabdus furcosa group* | Ambiguous taxa | 110 |
| Erysipelotrichales | Erysipelotrichaceae | *Allobaculum* | NA | 278 |
| Erysipelotrichales | Erysipelotrichaceae | *Allobaculum* | uncultured bacterium | 207 |
| Erysipelotrichales | Erysipelotrichaceae | *Dielma* | uncultured bacterium | 135 |
| Erysipelotrichales | Erysipelotrichaceae | *Dielma* | uncultured bacterium | 193 |
| Erysipelotrichales | Erysipelotrichaceae | uncultured | *Erysipelotrichaceae bacterium canine oral taxon 255* | 138 |
| Fusobacteriales | Fusobacteriaceae | *Fusobacterium* | Ambiguous taxa | 10 |
| Fusobacteriales | Fusobacteriaceae | *Fusobacterium* | Ambiguous taxa | 29 |
| Fusobacteriales | Fusobacteriaceae | *Fusobacterium* | uncultured bacterium | 184 |
| Fusobacteriales | Leptotrichiaceae | *Leptotrichia* | NA | 152 |
| Fusobacteriales | Leptotrichiaceae | *Leptotrichia* | NA | 174 |
| Fusobacteriales | Leptotrichiaceae | *Oceanivirga* | Ambiguous taxa | 214 |
| Fusobacteriales | Leptotrichiaceae | *Streptobacillus* | Ambiguous taxa | 115 |
| Fusobacteriales | Leptotrichiaceae | *Streptobacillus* | Ambiguous taxa | 159 |
| Ambiguous taxa | Ambiguous taxa | Ambiguous taxa | Ambiguous taxa | 126 |
| Ambiguous taxa | Ambiguous taxa | Ambiguous taxa | Ambiguous taxa | 144 |
| Gracilibacteria bacterium canine oral taxon 394 | Gracilibacteria bacterium canine oral taxon 394 | *Gracilibacteria bacterium canine oral taxon 394* | *Gracilibacteria bacterium canine oral taxon 394* | 139 |
| Gracilibacteria bacterium canine oral taxon 394 | Gracilibacteria bacterium canine oral taxon 394 | *Gracilibacteria bacterium canine oral taxon 394* | *Gracilibacteria bacterium canine oral taxon 394* | 145 |
| Gracilibacteria bacterium canine oral taxon 394 | Gracilibacteria bacterium canine oral taxon 394 | *Gracilibacteria bacterium canine oral taxon 394* | *Gracilibacteria bacterium canine oral taxon 394* | 31 |
| Burkholderiales | Burkholderiaceae | *Lautropia* | uncultured bacterium | 14 |
| Burkholderiales | Burkholderiaceae | *Lautropia* | uncultured bacterium | 175 |
| Burkholderiales | Comamonadaceae | *Corticibacter* | Ambiguous taxa | 149 |
| Burkholderiales | Comamonadaceae | *Corticibacter* | Ambiguous taxa | 69 |
| Burkholderiales | Comamonadaceae | NA | NA | 128 |
| Burkholderiales | Comamonadaceae | NA | NA | 229 |
| Burkholderiales | Comamonadaceae | *Ottowia* | Ambiguous taxa | 158 |
| Neisseriales | Neisseriaceae | *Aquaspirillum* | Ambiguous taxa | 70 |
| Neisseriales | Neisseriaceae | *Conchiformibius* | Ambiguous taxa | 109 |
| Neisseriales | Neisseriaceae | *Conchiformibius* | Ambiguous taxa | 246 |
| Neisseriales | Neisseriaceae | NA | NA | 408 |
| Neisseriales | Neisseriaceae | *Neisseria* | Ambiguous taxa | 39 |
| Neisseriales | Neisseriaceae | *Neisseria* | Ambiguous taxa | 81 |
| Desulfobacterales | Desulfobulbaceae | *Desulfobulbus* | Ambiguous taxa | 224 |
| Desulfovibrionales | Desulfovibrionaceae | *Desulfovibrio* | Ambiguous taxa | 219 |
| Campylobacterales | Campylobacteraceae | *Campylobacter* | *Campylobacter sp feline oral taxon 100* | 141 |
| Cardiobacteriales | Cardiobacteriaceae | Ambiguous taxa | Ambiguous taxa | 46 |
| Cardiobacteriales | Cardiobacteriaceae | NA | NA | 100 |
| Enterobacteriales | Enterobacteriaceae | NA | NA | 205 |
| Pasteurellales | Pasteurellaceae | *Frederiksenia* | NA | 16 |
| Pasteurellales | Pasteurellaceae | *Frederiksenia* | uncultured bacterium | 91 |
| Pasteurellales | Pasteurellaceae | *Haemophilus* | Ambiguous taxa | 333 |
| Pasteurellales | Pasteurellaceae | NA | NA | 235 |
| Pasteurellales | Pasteurellaceae | NA | NA | 645 |
| Pasteurellales | Pasteurellaceae | NA | NA | 99 |
| Pasteurellales | Pasteurellaceae | *Pasteurella* | Ambiguous taxa | 78 |
| Pasteurellales | Pasteurellaceae | uncultured | NA | 21 |
| Pseudomonadales | Moraxellaceae | *Acinetobacter* | NA | 52 |
| Pseudomonadales | Moraxellaceae | *Moraxella* | Ambiguous taxa | 1488 |
| Pseudomonadales | Moraxellaceae | *Moraxella* | Ambiguous taxa | 234 |
| Pseudomonadales | Moraxellaceae | *Moraxella* | Ambiguous taxa | 58 |
| Pseudomonadales | Moraxellaceae | *Moraxella* | Ambiguous taxa | 6 |
| Pseudomonadales | Moraxellaceae | *Psychrobacter* | NA | 19 |
| Pseudomonadales | Moraxellaceae | *Psychrobacter* | NA | 2 |
| Pseudomonadales | Pseudomonadaceae | *Pseudomonas* | Ambiguous taxa | 44 |
| Pseudomonadales | Pseudomonadaceae | *Pseudomonas* | *Pseudomonas koreensis* | 56 |
| Xanthomonadales | Xanthomonacadeae | *Luteimonas* | Ambiguous taxa | 156 |
| Xanthomonadales | Xanthomonacadeae | uncultured | NA | 28 |
| Ambiguous taxa | Ambiguous taxa | Ambiguous taxa | Ambiguous taxa | 117 |
| NA | NA | NA | NA | 267 |
| TM7 phylum sp canine oral taxon 237 | TM7 phylum sp canine oral taxon 237 | *TM7 phylum sp canine oral taxon 237* | *TM7 phylum sp canine oral taxon 237* | 232 |
| Unknown order | Unknown family | *Candidatus Saccharimonas* | NA | 314 |
| Unknown order | Unknown family | *Candidatus Saccharimonas* | *TM7 phylum sp canine oral taxon 363* | 137 |
| Spirochaetales | Spirochaetaceae | *Tremonema 2* | NA | 238 |
| Ambiguous taxa | Ambiguous taxa | Ambiguous taxa | Ambiguous taxa | 121 |
| Ambiguous taxa | Ambiguous taxa | Ambiguous taxa | Ambiguous taxa | 225 |
| Ambiguous taxa | Ambiguous taxa | Ambiguous taxa | Ambiguous taxa | 68 |
| Ambiguous taxa | Ambiguous taxa | Ambiguous taxa | Ambiguous taxa | 77 |
| Synergistales | Synergistaceae | *Fretibacterium* | Ambiguous taxa | 202 |
| Acholeplasmatales | Acholeplasmataceae | *Acholeplasma* | *Mycoplasma feliminutum* | 335 |
| Mycoplasmatales | Mycoplasmataceae | *Mycoplasma* | NA | 282 |
| Mycoplasmatales | Mycoplasmataceae | *Ureaplasma* | Ambiguous taxa | 212 |

Supplemental Table 2. Relative abundance (%) of predominant oral microbial taxa, present in canines during seven weeks of repeated salivary sampling. Relative abundance grouped by diet fed to canines during study.

|  | **Diet (± SD)** | | |  |
| --- | --- | --- | --- | --- |
| **Phylum** | **Chicken** | **Lamb** | **Salmon** | **P-Value** |
| Proteobacteria | 42.82 (21.73) | 40.1 (21.0) | 51.52 (20.76) | 0.7046 |
| Bacteroidetes | 25.26 (17.28) | 23.56 (15.88) | 16.5 (15.30) | 0.7046 |
| Firmicutes | 15.65 (8.12) | 22.71 (19.23) | 17.33 (10.97) | 0.7046 |
| Actinobacteria | 7.49 (8.13) | 4.58 (3.49) | 6.65 (6.35) | 0.7046 |
| Fusobacteria | 3.47 (3.96) | 4.32 (4.55) | 2.75 (3.78) | 0.7046 |
| Gracilibacteria | 1.93 (1.43) | 2.08 (1.72) | 2.15 (3.84) | 0.9568 |
| Absconditabacteria | 1.58 (1.80) | 1.35 (1.19) | 1.49 (1.50) | 0.9568 |
| Saccharibacteria | 1.49 (1.25) | 1.06 (1.39) | 1.38 (2.26) | 0.7046 |
| **Class** |  |  |  |  |
| Gammaproteobacteria | 33.31 (26.16) | 33.31 (26.16) | 43.95 (25.11) | 0.9967 |
| Flavobacteriia | 20.88 (14.89) | 18.8 (13.55) | 12.99 (12.46) | 0.9967 |
| Bacilli | 10.21 (8.53) | 17.42 (19.65) | 11.86 (9.22) | 0.9967 |
| Betaproteobacteria | 9.23 (5.99) | 6.56 (4.63) | 7.34 (5.70) | 0.9967 |
| Actinobacteria | 6.74 (7.53) | 3.99 (2.91) | 5.92 (5.80) | 0.9967 |
| Clostridia | 4.97 (4.26) | 4.71 (4.50) | 4.89 (4.62) | 0.9967 |
| Bacteroidia | 4.38 (5.62) | 4.75 (5.97) | 3.51 (7.57) | 0.9967 |
| Fusobacteriia | 3.47 (3.96) | 4.32 (4.55) | 2.75 (3.78) | 0.9967 |
| Absconditabacteria ambiguous taxa | 1.58 (1.80) | 1.35 (1.19) | 1.49 (1.50) | 0.9967 |
| Gracilibacteria bacterium canine oral taxon 394 | 1.1 (1.08) | 1.25 (1.11) | 1.42 (2.33) | 0.9967 |
| Saccharibacteria ambiguous taxa | 1.17 (1.06) | 0.68 (0.97) | 0.93 (1.74) | 0.9967 |
| **Order** |  |  |  |  |
| Flavobacteriales | 20.88 (14.89) | 18.8 (13.55) | 12.99 (12.46) | 0.8615 |
| Pseudomonadales | 19.36 (30.22) | 19.62 (27.0) | 30.78 (32.42) | 0.8615 |
| Pasteurellales | 9.16 (11.95) | 10.19 (9.93) | 9.64 (9.66) | 0.9967 |
| Lactobacillales | 8.39 (6.95) | 13.02 (14.99) | 9.55 (7.47) | 0.8615 |
| Burkholderiales | 6.84 (4.83) | 3.27 (2.56) | 4.11 (4.05) | 0.8615 |
| Clostridiales | 4.97 (4.26) | 4.71 (4.50) | 4.89 (4.62) | 0.9967 |
| Bacteroidales | 4.38 (5.62) | 4.75 (5.97) | 3.51 (7.57) | 0.9967 |
| Fusobacteriales | 3.47 (3.96) | 4.32 (4.55) | 2.75 (3.78) | 0.8647 |
| Neisseriales | 2.39 (1.95) | 3.28 (2.75) | 3.23 (3.65) | 0.8615 |
| Actinomycetales | 3.23 (4.71) | 1.67 (1.38) | 3.23 (4.85) | 0.8615 |
| Bacillales | 1.82 (3.70) | 4.4 (11.33) | 2.31 (2.56) | 0.8615 |
| Xanthomonadales | 2.57 (2.56) | 1.9 (1.71) | 1.65 (1.62) | 0.8615 |
| Cardiobacteriales | 2.22 (1.67) | 1.58 (1.70) | 1.52 (1.73) | 0.8647 |
| Absconditabacteria ambiguous taxa | 1.58 (1.80) | 1.35 (1.19) | 1.49 (1.50) | 0.9967 |
| Micrococcales | 1.69 (2.15) | 1.14 (1.61) | 1.78 (2.66) | 0.9821 |
| Gracilibacteria bacterium canine oral taxon 394 | 1.1 (1.08) | 1.25 (1.11) | 1.42 (2.33) | 0.9967 |
| Saccharibacteria ambiguous taxa | 1.17 (1.06) | 0.68 (0.97) | 0.93 (1.74) | 0.8615 |
| Corynebacteriales | 1.09 (1.12) | 0.72 (0.80) | 0.35 (0.53) | 0.8615 |
| **Family** |  |  |  |  |
| Flavobacteriaceae | 20.88 (14.89) | 18.8 (13.55) | 12.99 (12.46) | 0.8033 |
| Moraxellaceae | 18.05 (27.77) | 17.62 (23.98) | 30.77 (32.42) | 0.8033 |
| Pasteurellaceae | 9.16 (11.95) | 10.19 (9.93) | 9.64 (9.66) | 0.9777 |
| Streptococcaceae | 4.36 (3.07) | 7.07 (8.98) | 7.02 (6.48) | 0.8591 |
| Aerococcaceae | 3.66 (4.80) | 5.28 (6.60) | 2.26 (2.31) | 0.8033 |
| Porphyromonadaceae | 3.61 (4.62) | 4.13 (5.01) | 2.98 (6.18) | 0.9710 |
| Fusobacteriaceae | 3.03 (3.69) | 2.96 (3.48) | 2.37 (3.84) | 0.9777 |
| Actinomycetaceae | 3.23 (4.71) | 1.67 (1.38) | 3.23 (4.85) | 0.8033 |
| Burkholderiaceae | 3.85 (2.99) | 1.64 (1.54) | 2.56 (3.25) | 0.8033 |
| Neisseriaceae | 2.39 (1.95) | 3.28 (2.75) | 3.23 (3.65) | 0.8033 |
| Comamonadaceae | 3.0 (2.37) | 1.64 (1.47) | 1.55 (1.72) | 0.8033 |
| Xanthomonadaceae | 2.57 (2.56) | 1.9 (1.71) | 1.65 (1.62) | 0.8033 |
| Cardiobacteriaceae | 2.22 (1.67) | 1.58 (1.70) | 1.52 (1.73) | 0.8591 |
| Defluviitaleaceae | 1.97 (2.15) | 1.49 (1.52) | 1.31 (1.32) | 0.8768 |
| Absconditabacteria ambiguous taxa | 1.58 (1.80) | 1.35 (1.19) | 1.49 (1.50) | 0.9710 |
| Peptostreptococcaceae | 1.38 (1.12) | 1.36 (1.64) | 1.48 (1.89) | 0.9777 |
| Microbacteriaceae | 1.22 (1.54) | 0.58 (0.53) | 0.82 (0.76) | 0.8033 |
| Clostridiales Family XII | 1.21 (1.24) | 0.72 (0.90) | 1.19 (1.42) | 0.8567 |
| Pseudomonadaceae | 1.31 (7.39) | 2.0 (11.79) | 0.01 (0.03) | 0.9492 |
| Gracilibacteria bacterium canine oral taxon 394 | 1.1 (1.08) | 1.25 (1.11) | 1.42 (2.33) | 0.9710 |
| Corynebacteriaceae | 1.09 (1.12) | 0.72 (0.80) | 0.35 (0.53) | 0.8033 |
| Saccharibacteria ambiguous taxa | 1.17 (1.06) | 0.68 (0.97) | 0.93 (1.74) | 0.8033 |
| Bacillales family XI | 0.93 (1.66) | 2.04 (3.46) | 1.69 (3.46) | 0.8033 |
| Planococcaceae | 0.85 (3.53) | 2.09 (10.69) | 0.21 (0.96) | 0.8705 |
| Leptotrichiaceae | 0.44 (0.90) | 1.37 (2.66) | 0.38 (0.59) | 0.8033 |
| **Genus** |  |  |  |  |
| *Psychrobacter* | 10.94 (26.09) | 11.0 (25.93) | 25.72 (35.74) | 0.8077 |
| *Bergeyella* | 8.77 (7.78) | 11.32 (9.31) | 6.02 (6.51) | 0.8077 |
| *Streptococcus* | 4.36 (3.07) | 7.07 (8.98) | 7.02 (6.48) | 0.8077 |
| *Moraxella* | 4.72 (4.91) | 6.37 (5.93) | 5.03 (6.45) | 0.8077 |
| *Pasterurellaceae uncultured* | 4.61 (11.48) | 4.85 (8.63) | 5.37 (6.85) | 0.9922 |
| *Capnocytophaga* | 5.84 (4.26) | 3.87 (2.68) | 4.43 (5.74) | 0.8077 |
| *Flavobacterium* | 6.27 (7.12) | 3.61 (6.07) | 2.54 (3.42) | 0.8077 |
| *Porphyromonas* | 3.34 (4.38) | 3.81 (4.82) | 2.7 (5.58) | 0.9291 |
| *Frederiksenia* | 3.3 (2.61) | 3.56 (3.01) | 2.9 (3.71) | 0.9291 |
| *Fusobacterium* | 3.03 (3.69) | 2.96 (3.48) | 2.37 (3.84) | 0.9463 |
| *lautropia* | 3.85 (2.99) | 1.64 (1.54) | 2.56 (3.25) | 0.8077 |
| *Actinomyces* | 3.23 (4.71) | 1.67 (1.38) | 1.09 (1.12) | 0.8077 |
| *Abiotrophia* | 2.2 (4.68) | 4.42 (6.62) | 1.7 (1.72) | 0.8077 |
| *Xanthomonadaceae uncultured* | 2.56 (2.56) | 1.7 (1.45) | 1.63 (1.62) | 0.8077 |
| *Cardiobacteriaceae ambiguous taxa* | 2.2 (1.67) | 1.25 (1.21) | 1.18 (1.53) | 0.8077 |
| *Corticibacter* | 2.29 (1.93) | 1.04 (0.99) | 1.05 (1.48) | 0.8077 |
| *Defluviitaleaceae UGC-011* | 1.97 (2.15) | 1.49 (1.52) | 1.31 (1.32) | 0.8376 |
| *Acinetobacter* | 2.39 (11.57) | 0.24 (0.77) | 0.02 (0.07) | 0.8077 |
| *Neisseria* | 1.77 (1.55) | 1.78 (1.60) | 1.12 (1.16) | 0.8077 |
| *Absconditabacteria Ambiguous taxa* | 1.58 (1.80) | 1.35 (1.19) | 1.49 (1.50) | 0.9358 |
| *Aerococcoceae NA* | 1.46 (2.12) | 0.87 (0.95) | 0.56 (1.01) | 0.8077 |
| *Pseudomonas* | 1.31 (7.39) | 2.0 (11.79) | 0.01 (0.03) | 0.9037 |
| *Gracilibacteria bacterium oral taxon 394* | 1.1 (1.08) | 1.25 (1.11) | 1.42 (2.33) | 0.9291 |
| *Fusibacter* | 1.21 (1.24) | 0.72 (0.90) | 1.19 (1.42) | 0.8077 |
| *Saccharibacteria ambiguous taxa* | 1.17 (1.06) | 0.68 (0.97) | 0.93 (1.74) | 0.8077 |
| *Corynebacterium* | 1.09 (1.12) | 0.72 (0.80) | 0.35 (0.53) | 0.8077 |
| *Gemella* | 0.93 (1.66) | 2.04 (3.46) | 1.69 (2.40) | 0.8077 |
| *Leucobacter* | 1.05 (1.48) | 0.55 (0.50) | 0.75 (0.74) | 0.8077 |
| *Conchiformibius* | 0.2 (0.56) | 0.72 (1.55) | 1.62 (2.88) | 0.8077 |
